# Rethinking the Coral Microbiome: Simplicity Exists within a Diverse Microbial Biosphere

**DOI:** 10.1128/mBio.00812-18

**Published:** 2018-10-09

**Authors:** Alejandra Hernandez-Agreda, William Leggat, Pim Bongaerts, César Herrera, Tracy D. Ainsworth

**Affiliations:** a Australian Research Council Centre of Excellence for Coral Reef Studies, James Cook University, Townsville, Australia; b College of Public Health, Medical and Veterinary Sciences, James Cook University, Townsville, Australia; c School of Environmental and Life Sciences, The University of Newcastle, Ourimbah, Australia; d Global Change Institute, The University of Queensland, Brisbane, Australia; e California Academy of Sciences, San Francisco, California, USA; f TropWATER, Centre for Tropical Water & Aquatic Ecosystem Research, James Cook University, Townsville, Australia; g College of Science and Engineering, James Cook University, Townsville, Australia; h School of Biological, Earth and Environmental Sciences, University of New South Wales, Sydney, Australia; Max Planck Institute for Marine Microbiology

**Keywords:** bacteria, coral, holobiont, microbiome, symbiosis

## Abstract

Studies of the coral microbiome predominantly characterize the microbial community of the host species as a collective, rather than that of the individual. This ecological perspective on the coral microbiome has led to the conclusion that the coral holobiont is the most diverse microbial biosphere studied thus far. However, investigating the microbiome of the individual, rather than that of the species, highlights common and conserved community attributes which can provide insights into the significance of microbial associations to the host. Here, we show there are consistent characteristics between individuals in the proposed three components of the coral microbiome (i.e., “environmentally responsive community,” “resident or individual microbiome,” and “core microbiome”). We found that the resident microbiome of a photoendosymbiotic coral harbored <3% (∼605 phylotypes) of the 16S rRNA phylotypes associated with all investigated individuals of that species (“species-specific microbiome”) (∼21,654 phylotypes; individuals from *Pachyseris speciosa* [*n* = 123], *Mycedium elephantotus* [*n* = 95], and *Acropora aculeus* [*n* = 91] from 10 reef locations). The remaining bacterial phylotypes (>96%) (environmentally responsive community) of the species-specific microbiome were in fact not found in association with the majority of individuals of the species. Only 0.1% (∼21 phylotypes) of the species-specific microbiome of each species was shared among all individuals of the species (core microbiome), equating to ∼3.4% of the resident microbiome. We found taxonomic redundancy and consistent patterns of composition, structure, and taxonomic breadth across individual microbiomes from the three coral species. Our results demonstrate that the coral microbiome is structured at the individual level.

## Abstract (precis)

We propose that the coral holobiont should be conceptualized as a diverse transient microbial community that is responsive to the surrounding environment and encompasses a simple, redundant, resident microbiome and a small conserved core microbiome. Most importantly, we show that the coral microbiome is comparable to the microbiomes of other organisms studied thus far. Accurately characterizing the coral-microbe interactions provides an important baseline from which the functional roles and the functional niches within which microbes reside can be deciphered.

## INTRODUCTION

Deciphering the functional contribution of symbiotic bacteria to host health is imperative to determine the mechanistic basis of coral health, survival, and resilience in a rapidly changing environment. However, to date, the challenge remains to understand which of the thousands of bacteria that are in association with a particular coral host species (“species-specific microbiome”) have a significant contribution to the well-being of individual corals in their natural habitat (1–3). Accurately documenting the taxonomic structure of the coral microbiome has been crucial to this aim, and over the past decade, numerous studies have aimed to define the characteristics of the healthy coral microbiome through the use of amplicon sequencing (2, 4). Importantly though, it is the interactions of the individual with its microbiome (“individual microbiome”) that impact coral health. Distinguishing the individual’s microbiome from that of species-specific microbial consortia is therefore critical to identify the symbiotic microbial roles.

As a holobiont or meta-organism, corals are inhabited by diverse microbial communities including microalgae, bacteria, fungi, archaea, and viruses (5). The endosymbiosis of coral with the zooxanthellae *Symbiodinium* is, so far, the most studied and best understood biological interaction between coral and any member of the microbial community (6). The coral relies on this symbiotic relationship with the dinoflagellate for up to 95% of its nutrition uptake (7, 8), and when this endosymbiosis is disrupted for long periods of time by environmental stress, the coral can perish (9–11). However, for all other members of the microbial community, the stability and functional contribution of the host-microbe interaction have largely remained unknown. One of the main factors limiting our understanding of these bacterial associations is the variability that is evident in community associations (12, 13), whereby determining conserved functional contributions by specific bacteria has remained elusive. Coral-associated bacterial communities are highly variable, diverse, and abundant, and differentiating the stable, functionally significant interactions within these communities is challenging (4). Variability in the bacterial community is known to occur at different spatial and temporal scales, including within the many microbial niches of a coral colony, at a scale of centimeters (13), and along different biogeographical regions, across a scale of thousands of kilometers (14–16). Thus, corals represent a variable ecosystem and host habitat for bacterial associations.

**FIG 1:**
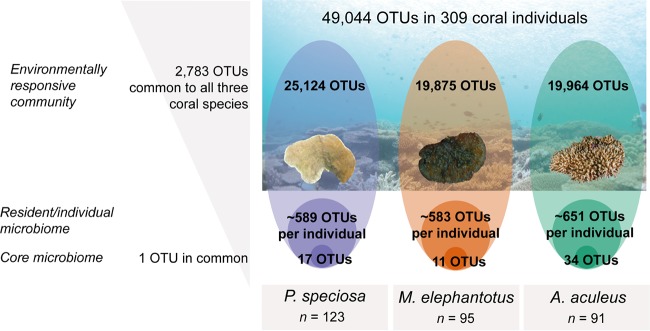
Coral microbiome conceptualized into three distinct layers. We propose that the microbiome of each coral individual should be categorized into three distinct groups of bacteria with different levels of impact from the environment and host: (i) an environmentally responsive community with thousands of bacterial phylotypes, transient and highly variable across coral individuals; (ii) individual microbiome, ∼500 to 600 OTUs that vary among reefs at the level of OTUs but consistently belong to three major taxonomic classes; and (iii) core microbiome, few bacterial phylotypes, potentially symbiotic. Taxonomic (and potentially functional) redundancy is occurring in the resident community and the core microbiome. The reef picture was provided by Alexander J. Fordyce.

However, here we argue that the diversity, structure, and potential function of coral bacterial associations are much lower and less complex than has previously been reported (5). We investigated the common and conserved attributes of the healthy coral microbiome using 309 individuals collected from three highly abundant and widespread Indo-Pacific species (17), *Acropora aculeus*, *Mycedium elephantotus*, and *Pachyseris speciosa*. We applied bacterial 16S rRNA gene amplicon sequencing to examine the bacterial associations of each coral specimen, collected from 10 reefs from central and northern parts of the Great Barrier Reef and the Coral Sea, at depth intervals of 10, 20, 40, and 60 to 80 m. We propose that the coral microbiome can be divided into three distinct layers (Fig. 1): (i) the “environmentally responsive community,” which is predominantly a transient community encompassing thousands of distinct 16S rRNA phylotypes, of which very few are associated with a single host individual; (ii) the “resident community” consisting of phylotypes principally from three key bacterial classes, *Alpha*- and *Gammaproteobacteria* and *Deltaproteobacteria* in *M. elephantotus* or *Flavobacteriia* in *P. speciosa* and *A. aculeus*; and (iii) the “core microbiome” consisting of a few ubiquitous, potentially symbiotic, bacterial phylotypes. The identity of phylotypes composing the coral core microbiome depends on, among other factors, the coral species considered (4).

## RESULTS AND DISCUSSION

**FIG 2:**
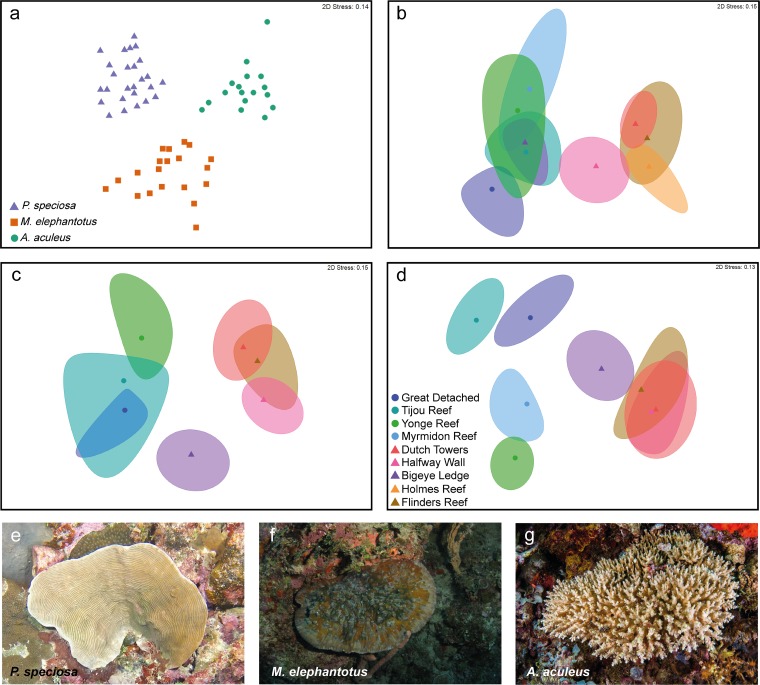
Bacterial community structure differed spatially and between coral species. Nonmetric multidimensional scaling (nMDS) based on relative abundance to illustrate differences between coral species (a) (*P < *0.01 by PERMANOVA [see Table S4 at https://figshare.com/s/ffadfdf1a1bab8a37088]) and between reefs for *P. speciosa* (b and e), *M. elephantotus* (c and f; excluding Myrmidon reef), and *A. aculeus* (d and g; excluding Holmes reef). nMDS based on Bray-Curtis dissimilarity of fourth-root-transformed data. (a) Centroids, (b to d) bootstrap area and average for reefs. Circles denote Great Barrier Reef (GBR) reefs, and triangles indicate Coral Sea (CS) reefs. For presence/absence equivalent results, see Fig. S1 at https://figshare.com/s/ffadfdf1a1bab8a37088. The *A. aculeus* photo was provided by Ed Roberts.

**FIG 3:**
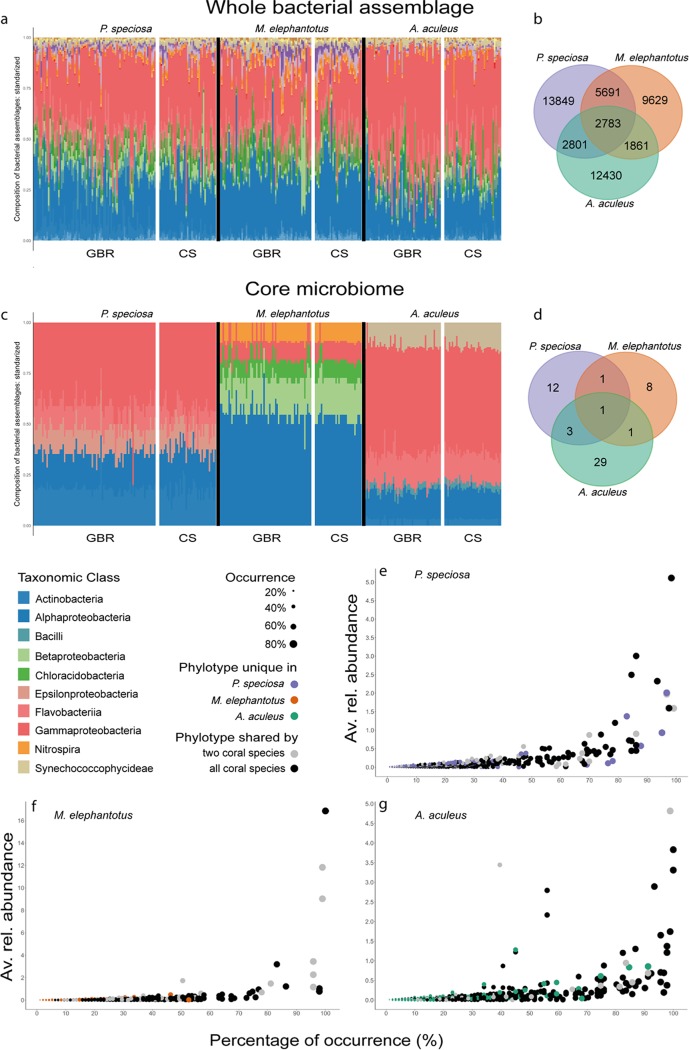
The coral microbiome comprises common and species-specific phylotypes in a stable taxonomic structure across individuals. The taxonomic structure of coral-associated bacteria within individual coral hosts (a) was reflected by that of the core microbiome (c). *Alpha*- and *Gammaproteobacteria* dominated the bacterial assemblage composition despite the variability across spatial scales (Fig. 2). Bacterial classes were structurally stable across individuals. Common bacterial phylotypes of the three commonly occurring coral species (species-specific microbiome and resident microbiome [b]) and core microbiome of individual *P. speciosa*, *M. elephantotus*, and *A. aculeus* corals (d). Graphs of the average relative abundance versus percentage of occurrence across coral individuals for *P. speciosa* (e), *M. elephantotus* (f), and *A. aculeus* (g) revealed that highly persistent (≥80%) OTUs are rarely species specific (note the difference in scale in relative abundance). Bacterial assemblage composition was defined by the number of OTUs belonging to the taxonomic level class, standardized by the total per individual. For the extended taxonomic legend, see Fig. S16 at https://figshare.com/s/ffadfdf1a1bab8a37088.

Across ecological systems, the concept of taxonomic and functional redundancy has been employed to characterize and conceptualize healthy and disturbed ecosystem states (18–21). Here, we investigated the microbiomes of 309 coral individuals from three highly abundant and widespread Indo-Pacific species (17), the plating corals *Mycedium elephantotus* and *Pachyseris speciosa* and the branching coral *Acropora aculeus.* The diversity and species-specific patterns of the microbiome observed for these three species were consistent with previous studies (5), in that collectively, corals host highly diverse bacterial interactions which are responsive to the hosts’ reef environment (Fig. 2). We reported more than 79,000 distinct operational taxonomic units (OTUs) generated from 17 million sequences within the collective data set (see Table S1 at https://figshare.com/s/ffadfdf1a1bab8a37088), which resulted in 49,000 distinct OTUs (from ∼6 million sequences) after filtering chloroplasts and unidentified/unassigned (not assigned to Kingdom *Bacteria*) sequences. From those OTUs, about half were species specific, and half were shared between the three coral species (Fig. 3b; see also Table S1), with each coral species hosting on average ∼20,000 to 25,000 OTUs (19,964 OTUs for *M. elephantotus*, 25,124 OTUs for *P. speciosa*, and 19,875 OTUs for *A. aculeus*). Importantly, we found that each individual coral hosts very few of these species-specific bacterial phylotypes. For example, *P. speciosa* individuals hosted on average only 589 ± 39 OTUs (*n* = 123 coral individuals); similarly, on average, *M. elephantotus* individuals hosted 583 ± 62 OTUs (*n* = 95 corals) and *A. aculeus* individuals hosted 651 ± 41 OTUs (*n* = 91 corals). As such, regardless of the coral species, the reef site in which they reside, or other environmental variables (such as reef depth and nutrient and light availability), an individual coral colony harbors only 2 to 3% of the total number of bacteria that are found in association with the species (species-specific microbiome). We also found that the bacterial associations of individual corals are overwhelmingly constrained to two or three dominant bacterial classes (Fig. 3a and c). These findings are consistent with the characteristics of the microbiome of holobiont models across the phyla that have been studied thus far, including hydra (22, 23), nematode Caenorhabditis elegans (24), plants (25, 26), and humans (20, 27), which similarly identified that the host microbiome is highly structured, habitat specific, and functionally redundant. As such, our findings contrast with previous studies hypothesizing that the coral microbiome is as an exceptionally diverse microbial biosphere compared to other organisms. As it has been widely documented and further supported by this study, corals host thousands of bacteria in species-specific interactions. However, these high numbers of species-specific interactions are due to a highly transient microbiome (Fig. 2), likely reflective of the highly dynamic symbiotic state and open interaction with the surrounding environment. We further suggest that the substantial taxonomic redundancy within an individual coral’s microbiome may reflect functional redundancy of the highly efficient photoendosymbiotic host system, particularly within waste production, utilization, and nutrient cycling.

### Resident community.

The composition of the bacterial assemblages observed in individual corals, based on the number of OTUs per bacterial class, revealed that *Alpha*- and *Gammaproteobacteria* are consistently the dominant taxonomic classes across coral species, geographic regions, and depth (Fig. 3). Here, we report that the numerical dominance of phylotypes within these two bacterial classes, which was reflected in the taxonomic structure, was similar to that which has been previously reported in the numerical abundance of phylotypes within the *Alpha*- and *Gammaproteobacteria* (2, 4, 5). Thus, we show that a clear taxonomic redundancy was evident within the individual coral’s microbial assemblage. This pattern of constrained diversity was even more evident when evaluating beta-diversity and the indices of complexity to assess the microbiome.

**FIG 4:**
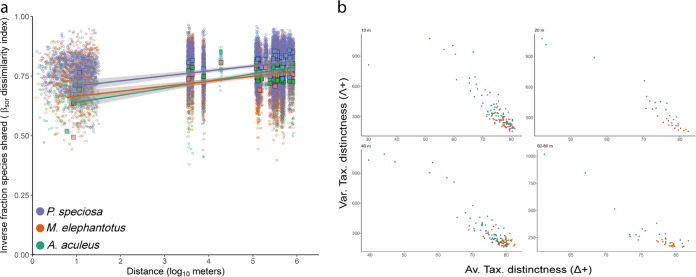
Beta-diversity (turnover [a]) and taxonomic breadth (b) of bacterial assemblages. (a) A minimal increase on dissimilarity (i.e., the inverse fraction of species shared) was observed between pairs of individuals across space or distance. Depending on the spatial scales, the composition of the coral microbiome was conserved in 50 to 23% (same reef) and 27 to 18% (distinct reefs). (b) The pattern of high taxonomic relatedness and low taxonomic evenness in the three species regardless of the depth supports the taxonomic structure observed in the coral microbiome. Together, these results suggest that a fraction of the coral microbiome is conserved and taxonomically structured, regardless of the reef environment.

Variations in the composition of bacterial assemblages (beta-diversity [turnover]) (28) showed that the bacterial distance-decay relationship for the three coral species is constant across space or distance, which is a unique feature of the coral microbiome compared to other ecological systems (29–31) (Fig. 4a). This indicates that regardless of community drivers related to the reef environment, the composition of the coral microbiome system is structured at the individual level. The structure and complexity of the assemblage, expressed as taxonomic relatedness (average of taxonomic distinctness [Δ^+^]) (32) and taxonomic evenness (variation of taxonomic distinctness [Λ^+^]) (33), provide a measure of the taxonomic spread of communities (34) which has been applied in macroecology (35–37) and more recently utilized in microbial ecology (38, 39). This allows us to assess biodiversity changes on spatial and temporal scales and the response to disturbances. By utilizing this approach in the current study, we found that the taxonomic relatedness is inversely proportional to the taxonomic evenness, thus indicating that the individual microbiome with high taxonomic complexity (high average, Δ^+^) are more even (low variance, Λ^+^) and vice versa. This pattern was consistent across all individuals of the three coral species studied, at all depths evaluated, and across all reef locations (Fig. 4b). Interestingly, both *M. elephantotus* and *P. speciosa* showed a constrained range in both average and variation of complexity beyond 20 m in reef depth (see Fig. S2 to S6 at https://figshare.com/s/ffadfdf1a1bab8a37088). In contrast, *A. aculeus* exhibited a broad response ranging across all reef depths. These results suggest that coral growth form, branching versus plating and/or massive forms, may have a substantial influence over the complexity and variation of the coral microbiome, particularly across reef depths.

### Core microbiome.

**FIG 5:**
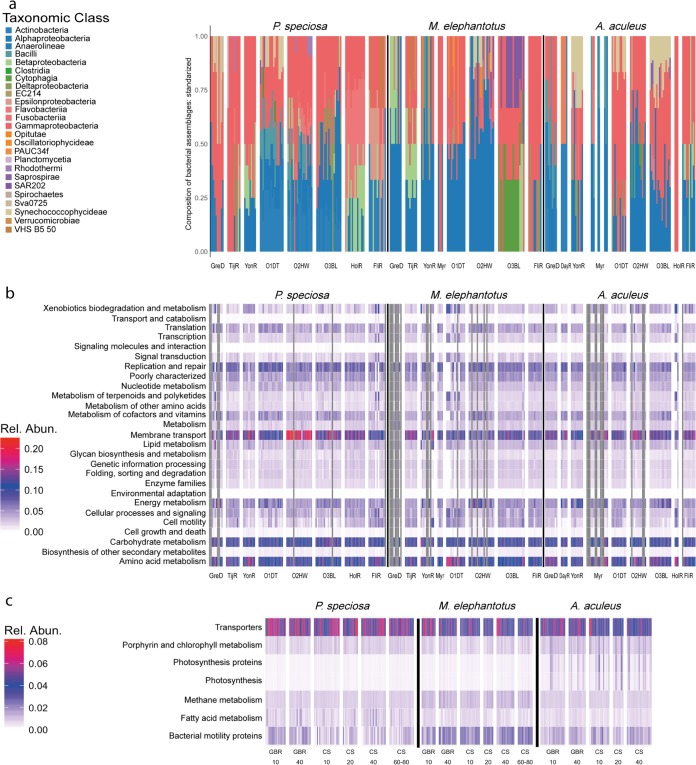
Different bacterial taxonomic structure of representative and highly persistent OTUs (core microbiome) with similar functional capabilities. For each coral species, representative OTUs (a) and core microbiome had a distinct taxonomic structure (Fig. 3c), but equivalent prediction on functional content (b and c). Functional prediction content generated from the relative abundance of KEGG Orthology (KO) genes, normalized and standardized by the total per individual.

A key feature of the individual microbiome in all three corals species studied was the characteristically small group of highly persistent OTUs (core microbiome) (Fig. 3c to g). Each coral species’ core microbiome encompassed phylotypes ranging from rare to highly dominant across reef habitats. In addition, very few bacterial phylotypes were shared between the three coral species. We also found similarities in the taxonomic structure of the core bacteria across species and individuals (core microbiome herein defined as when a bacterial species is present in ≥80% individuals within the study [Fig. 3c and d]). In each coral species, the core microbiome was equivalent in taxonomic complexity and predicted functional capabilities (Fig. 5c; see also Fig. S13 to S15 at https://figshare.com/s/ffadfdf1a1bab8a37088).

The core microbiome of both *P. speciosa* and *A. aculeus* was dominated by *Gammaproteobacteria* phylotypes and included several phylotypes with relative abundance between 0.43 and 5.11% and 0.15 to 4.82%, respectively (Fig. 3e to g; see also Table S2 at https://figshare.com/s/ffadfdf1a1bab8a37088). Overall, the core microbiome of *P. speciosa* and *A. aculeus* showed similar taxonomic structure (Fig. 3c), as observed in the bacterial assemblage of the species as a whole (Fig. 3a), with high numbers of phylotypes from *Alpha*- and *Gammaproteobacteria*. The *P. speciosa* core microbiome included phylotypes of the genera *Acinetobacter*, *Cloacibacterium*, *Corynebacterium*, *Gluconacetobacter*, *Mycobacterium*, *Pseudoalteromonas*, *Propionibacterium*, *Pseudomonas*, and *Rhodobacter* and family *Hyphomicrobiaceae*. The *A. aculeus* core microbiome comprised OTUs from the genera *Alicyclobacillus*, *Alteromonas*, *Oleibacter*, *Prochlorococcus*, *Propionibacterium*, *Pseudoalteromonas*, *Pseudomonas*, *Synechococcus*, and *Vibrio* and families *Endozoicomonadaceae* and *Flavobacteriaceae*. Interestingly, the core microbiome of the coral *M. elephantotus* was unique compared to the core microbiomes of the other two corals. Most notably, we found that the *Alphaproteobacteria* are the dominant group within the *Mycedium* microbiome (Fig. 3a), followed by *Betaproteobacteria*. Furthermore, in *M. elephantotus*, the most dominant 11 phylotypes each accounted for a relative abundance between 0.78 and 16.85% (≥80% occurrence in Fig. 3f; see also Table S2 at https://figshare.com/s/ffadfdf1a1bab8a37088). Moreover, *M. elephantotus* hosted three phylotypes with a high relative abundance (>9%) which together accounted for 37.7% of the relative abundance. These phylotypes belong to the classes *Alphaproteobacteria*, *Betaproteobacteria*, and *Chloracidobacteria*; family Ellin6075, and orders EC94 and *Kiloniellales*. The family Ellin6075 (class *Chloracidobacteria*) is unusual in the coral microbiome, but it has been previously found, with variable abundance, in microbialites (40) and soil (41, 42). Members of this family possess a nitrifying capacity (43).

The three coral species have common and distinct biomechanical, morphological, reproductive, and ecological traits. They are all of colonial morphology attached to the benthos, broadcast spawners, are hermaphrodites, and coincide in their *Symbiodinium* clade association (C3 and C3h in common) (44). However, they also have distinctive traits that may potentially influence microbial associations. *M. elephantotus* belongs to a robust major clade with encrusting and laminar growth forms that have large polyps (7.5 to 10 mm). *P. speciosa* and *A. aculeus* belong to the complex clade, have small polyps (0.8 to 1 mm and 2.4 to 4.6 mm, respectively) and are variable in growth forms (corymbose and laminar, respectively) (44). While the relationship between some of these traits and the coral microbial community has been explored (morphology [45, 46], phylogeny [45], mode of larval development [47–50], zooxanthellae clade [51]), to date, no direct or conclusive relationships have been drawn.

In the current study, only one phylotype, a member from the family *Alteromonadaceae* (OTU 806717), was found to be highly persistent in all three depth generalist coral species across the entire 10 to 80 m depth range (Fig. 3d). This bacterial phylotype was not only highly persistent in that it was present in 98.4% of individuals, but it was also present at a high relative abundance within both *A. aculeus* and *P. speciosa* individuals. This is the first report of a persistent bacterial phylotype within this group across coral species and a broad range of geographical and depth reef locations. However, members of the family *Alteromonadaceae* have been previously reported to occur in the core microbiome of other coral species (52) and in early life stages of coral (50, 53) and to be important in chemical defenses against pathogenesis in crustaceans (54). A search comparison of the sequence of this phylotype in the BLAST Nucleotide database (National Center of Biotechnology Information [NCBI] [see Table S3 at https://figshare.com/s/ffadfdf1a1bab8a37088]) showed close affiliation to an uncultured bacterium, a member of the *Symbiodinium* core microbiome (55), and to *Alteromonas* sp. and *Alteromonas macleodii*, which are bacteria found to coexist with cyanobacteria *Trichodesmium* and *Prochlorococcus* (56–58). When associated with *Prochlorococcus*, the opportunistic copiotroph and heterotroph bacteria of the genus *Alteromonas* degrade algal polysaccharides and remove reactive oxygen species (ROS) (56, 59, 60). The persistence of this specific taxon (OTU 806717) could indicate that it plays a critical functional role for the coral or for the coral-zooxanthellae symbiosis. The potential role of members of the *Alteromonadaceae* family in association with corals has yet to be explored.

### Environmentally responsive community.

Given that the reef environment varies across the depth gradient (61) and abiotic factors within the reef impact nutrient acquisition and cycling within the coral host (62, 63), these are also likely to be drivers of microbiome structure. Therefore, we also aimed to determine whether bacterial associations were reflective of the locations within the reef (depth of sampling) from which the corals were collected. We identified bacteria present within all host corals at various depths for each reef location. All three coral species showed persistent bacterial phylotypes across the sampled depths (see Fig. S7 at https://figshare.com/s/ffadfdf1a1bab8a37088). However, at all depths, the *A. aculeus* microbiome harbored the greatest diversity and highest number of phylotypes. Interestingly, the family *Endozoicomonadaceae* was present in the core microbiome of *A. aculeus* at all sampled depths, but was present in *M. elephantotus* and *P. speciosa* corals only occasionally. The family *Endozoicomonadaceae* was found in some *M. elephantotus* individuals collected at 10 m (TijR) and 40 m (FliR) and in *P. speciosa* individuals at 10 m (MyrR, O1DT), 40 m (O3BL), and 60 to 80 m (O2HW). Similarly, phylotypes from the phylum *Cyanobacteria*, including the genera *Synechococcus* and *Prochlorococcus*, were present in all individuals of the coral *A. aculeus* at all depths and reefs, but only occasionally in *M. elephantotus* (10 m at O1DT and O3BL, 20 m at O2HW, 40 m GreD and TijR, and 60 to 80 m at O1DT) and *P. speciosa* (all depths at TijR and YonR, 10 m at O1DT and O3BL, 40 m at MyrR, and 60 to 80 m at O2HW, O3BL, and FliR). This clear structuring of the coral microbiome was evident across the biogeographical and depth ranges of the current study, highlighting that redundancy is a consistent feature of the coral microbiome regardless of the region, site, and depth of the reef.

Significantly, analyses over the different spatial scales and depth gradients of the 309 coral individuals examined in the current study showed that the number of sequences and phylotypes and taxonomic structure of depth generalist corals *P. speciosa*, *M. elephantotus*, and *A. aculeus* were clearly comparable (see Fig. S8 to S12 and Table S4 at https://figshare.com/s/ffadfdf1a1bab8a37088). While we found differences in the microbiome in geographic regions of the three coral species (Great Barrier Reef [GBR] versus the Coral Sea [CS]; *P < *0.05 by permutational multivariate analysis of variance [PERMANOVA]), differences between reef localities (within region; *P < *0.05 by PERMANOVA; see Tables S5 to S8) and depths were not consistent across species (*a posteriori* analyses, Fig. 2; see also Tables S9 to S12). For example, we found that at Halfway Wall, Bigeye Ledge (Osprey), and Flinders of the Coral Sea, the bacterial assemblages within these reef localities showed similar assemblage structures at 10 m and 20 m but unique assemblages at 40 m and 60 to 80 m. Likely reflective of changes associated with mesophotic conditions (in this case 40 m and deeper), these results suggest substantial variation between individuals and between environments which have different abiotic drivers (such as light penetration, shading, nutrient availability, and water flow). This is highly likely within the diverse habitats of any coral reef environment whereby corals may be exposed to greater upwelling, flow, or shading depending on local reef factors, all of which are known to affect the physiology of the coral, its nutrient cycling, and its reliance on heterotrophic and autotrophic feeding (61–65).

### Taxonomic redundancy.

Finally, representative phylotypes from the transient community in each reef for each coral species were selected as groups of OTUs with a multivariate pattern, reflective of that observed in the species-specific microbiome (BVSTEP algorithm, ρ > 0.95). We then evaluated taxonomic relatedness and conducted functional prediction analysis for each individual coral. We found that taxonomic redundancy is reflected in the functional predictions for the corals' microbiome (Fig. 5a and b). We also found that the core microbiome and representative phylotypes per reef were equivalent in taxonomic complexity (Δ^+^ and Λ^+^, Fig. 3c and Fig. 5a; see also Table S13 at https://figshare.com/s/ffadfdf1a1bab8a37088) and in functional predictions (Fig. 5b and c; see also Fig. S13 to S15). This research clearly demonstrates that regardless of the bacterial assemblages at a reef, they are taxonomically, and likely functionally, redundant within the coral host. For example, transporters, porphyrin and chlorophyll metabolism, photosynthesis proteins, photosynthesis, methane metabolism, fatty acid metabolism, and bacterial motility proteins were consistently enriched within the coral-associated bacterial assemblage of individual corals across reef habitats (see level 3 KEGG Orthology (KO) at https://figshare.com/s/ffadfdf1a1bab8a37088). This is likely to indicate that within the nutritionally dynamic coral host, there are relatively few niche microhabitats available (e.g., gut [66], surface mucous layer [67], and skeleton [68]) and that those niches are highly consistent across species and reef environments. The hypothesis of functional redundancy in the coral microbiome has yet to be tested.

### Conclusion.

We find that the observed simplicity and structure of the corals’ diverse microbial biosphere, in which there is substantial taxonomic (and potentially functional) redundancy, is consistent across coral species, bioregions, and reef depths. These results thereby suggest that within diverse microbial biospheres, such as coral, simplicity exists despite complex environmental drivers. For the individual coral meta-organisms’ microbiome, this simplicity is on average 605 bacterial phylotypes and is likely to be reflective of coral microhabitats and the processes occurring on them. Conceptualizing the coral microbiome as a system of transient, resident, and core microbiomes is likely to make deciphering the microbial contribution to corals’ symbiotic and dysbiotic states more achievable.

## MATERIALS AND METHODS

Fragments of corals *Mycedium elephantotus* (*n = 95*) and *Acropora aculeus* (*n = 91*) were collected from 10 reefs from northern reefs of the Great Barrier Reef (GBR) and the Coral Sea (CS) during the Caitlin Seaview Survey expeditions. Coral specimens were collected at shallow and intermediate depth (10 to 40 m) using regular open-circuit diving between September and December 2012. At mesophotic depth (60 to 80 m), coral specimens were sampled in November 2013 using a remotely operated vehicle (ROV). Based on the bathymetric distribution of the coral species (61), between three and eight individuals were collected at each depth, from each reef location, and samples were preserved in salt-saturated 20% dimethyl sulfoxide (DMSO) with 0.5 M EDTA and stored at –20**°**C. A nested hierarchical design was used for collection and data analysis: (i) coral species, (ii) region (fixed factor, two levels: GBR and CS), (iii) reefs (random factor nested in region, 10 levels: for the GBR, Great Detached, Tijou Reef, Day Reef, Yonge Reef, and Myrmidon Reef; for the CS, Flinders Reef, Holmes Reef, and in Osprey Reef Dutch Towers, Halfway Wall [also known as Nautilus Wall], and Bigeye Ledge); and (iv) depth [fixed factor, four levels: 10 m (±3 m), 20 m (± 2 m), 40 m (±3 m), and 60 to 80 m]. Coral specimens were collected under permits supplied by the Great Barrier Reef Marine Park Authority (Townsville, Australia) and Commonwealth Marine Reserves, Department of the Environment (Hobart, Australia). For coordinates and site information of reef localities, see references 69 and 61.

### DNA extraction, amplification, and sequencing.

DNA extraction was performed using 0.4 (±0.02) g of each coral fragment using the modified protocol of MoBio PowerPlant pro DNA isolation kit (catalog no. 13400-50; MoBio, Carlsbad, CA) described by S. Sunagawa et al. (45). DNA concentration was estimated in a Qubit fluorometer using the Qubit dsDNA (double-stranded DNA) HS (high-sensitivity) assay kit (Thermo Fisher Scientific, Wilmington, DE). Amplified DNA was stored at −20°C before PCR amplification. Genomic template primers 27F/519R (v1-v3 region) were used to amplify bacterial 16S rRNA gene amplicons for examining bacterial assemblage structure. Gene amplicons were amplified in a single-step, 30-cycle PCR (HotStarTaq plus master mix kit; Qiagen, USA). The conditions for PCR were as follows: (i) 3 min at 94°C; (ii) 28 cycles, with each cycle consisting of 30 s at 94°C, 40 s at 53°C, and 1 min at 72°C; (iii) a final elongation step of 5 min at 72°C. PCR products were checked in 2% agarose gels, and samples were pooled in equal proportions based on molecular weight and DNA concentrations. Pooled samples were purified using calibrated Ampure XP beads. DNA library was prepared following the Illumina TruSeq DNA library protocol. Sequencing was performed by MR DNA (Molecular Research LP; Shallowater, TX, USA) using 300-bp paired ends on an Illumina MiSeq platform following the manufacturer’s guidelines.

### Sequence analysis.

To establish common phylotypes among coral species, *M. elephantotus* and *A. aculeus* data (newly generated here) were jointly analyzed with *P. speciosa* sequence data (previously analyzed in reference 69). Sequence data analysis was performed using the open-source software Quantitative Insights Into Microbial Ecology (QIIME, version 1.9) (70). Sequences with ambiguous base calls, with homopolymer runs exceeding 6 bp or below 200 bp, were discarded. Barcodes, primers, and chimeras were removed from sequences prior to analysis (Usearch61 [71] for chimera removal). Operational taxonomic units (OTUs) were defined and taxonomically identified with 97% cluster similarity using the RDP classifier and Greengenes database (version 13_8 [72]).

### Statistical analysis, taxonomic redundancy, and core microbiome.

Data mining and statistical and taxonomic redundancy analyses were performed with PRIMER v7 and PERMANOVA+ (73). Bacterial assemblage structure was analyzed by composition and structure. Abundance matrices were normalized using the fourth root transformation and standardized by the total per sample. Fourth root transformation was selected to balance the contribution of rare and highly abundant bacteria (74). Raw abundance OTU tables were also converted to presence/absence data to analyze bacterial composition. For both relative abundance and composition data, significant differences in the factors of the design were identified by permutational multivariate analysis of variance (PERMANOVA) using 9,999 permutations, Bray-Curtis and weighted Unifrac distances (on relative abundance data), and Sorensen and unweighted Unifrac distances (on composition data). All the sequences and OTUs obtained from specimens of a species were considered the species-specific microbiome or the whole microbiome of that coral species. Coral species data were analyzed separately after detection of structural differences between them (i.e., excluding the factor “coral species” from statistical analysis). Pairwise comparisons were used for further exploration of significant differences for any factor. PERMANOVA results were visualized in nonmetric multidimensional scaling (nMDS) (75) plots using 95% bootstrap reefs or centroids (identified in figure legends).

A stepwise selection of species (BVSTEP routine) was used to create subset matrices of selected OTUs reflecting the abundance pattern observed in the normalized relative abundance matrix (76–78). Using Bray-Curtis distance matrices, Spearman rank as the correlation method and Rho (ρ) of >0.95 and Delta Rho of 0.001 as the stop criteria, BVSTEP was run for each reef per coral species. Taxonomic redundancy and functional prediction were evaluated on the resulting subset matrices and the core microbiome for each coral species.

Phylotypes consistently present in ≥80% of the individuals were considered highly persistent core microbiome (79). Core 80% was identified for (i) each coral species (ii) each reef per coral species; and (iii) each depth per reef per coral species using the command compute_core_microbiome.py in QIIME. Core 80% matrices were created as subset matrices selecting core 80% OTUs from the normalized relative abundance matrix. Taxonomic redundancy was evaluated using the indices average [Δ+] (32) and variation [Λ+] (33) of taxonomic distinctness. Venn diagrams for the core 80% and the whole bacterial community were generated using Venn diagram software (Bioinformatics and Evolutionary Genomics [http://bioinformatics.psb.ugent.be/webtools/Venn/]). A search in the BLAST Nucleotide database (National Center of Biotechnology Information [NCBI] [https://blast.ncbi.nlm.nih.gov/]) was conducted for the sequence of the OTU 806717 (family *Alteromonadaceae*) to identify the closest sequence affiliation. Based on the percentage of identity and E value, the top 20 significant alignments were selected and presented in Table S3 at https://figshare.com/s/ffadfdf1a1bab8a37088.

### Predicted functional profiling based on bacterial taxonomy.

The Galaxy web version of Phylogenetic Investigation of Communities by Reconstruction of Unobserved States (PICRUSt [80]) was used to produce a prediction of the metagenomic functional content of the subset matrices of representative phylotypes and the core 80% matrices. Each matrix was normalized by copy number, and the metagenome prediction was produced using KEGG Orthology (KOs) and summarized at levels 2 and 3 of KEGG Pathway. The predicted functional profiles of representative OTUs in *P. speciosa* individuals from Myrmidon reef were not estimated since the OTUs were not present in the Greengenes database. Differences in KEGG pathways were assessed as indicated previously (see “Statistical analysis, taxonomic redundancy, and core microbiome” above). Graphs were produced with ggplot2 (81) as implemented in R (82).

### Beta-diversity (turnover).

Beta-diversity was analyzed using packages betapart (28, 83, 84) and geosphere in R (82). Pair-wise turnover was computed for each presence/absence OTU table using Sorensen dissimilarity index. Distance matrixes (one per coral species) among pairs of samples were calculated using their geographic locations and the Vincenty ellipsoid method. Due to the impossibility of assigning the exact location within the reef, we assigned a random location to each colony sampled in each reef (3 to 33 m from the reef coordinate). The relationship between the fraction of species shared (i.e., turnover) and distance for each pair of samples per coral species was explored in plots produced with ggplot2 (81) in R (82).

### Data availability.

Raw sequences are available in the National Center for Biotechnology Information (NCBI) Sequence Read Archive (SRA) under project number PRJNA435628, accession numbers SAMN10088816 to SAMN10089001.
